# The Germline-Restricted Chromosome of Male Zebra Finches in Meiotic Prophase I: A Proteinaceous Scaffold and Chromatin Modifications

**DOI:** 10.3390/ani14223246

**Published:** 2024-11-12

**Authors:** Sergey Matveevsky

**Affiliations:** Vavilov Institute of General Genetics, Russian Academy of Sciences, 119991 Moscow, Russia; sergey8585@mail.ru or s.matveevsky@vigg.ru

**Keywords:** germline-restricted chromosome, *Taeniopygia guttata*, synaptonemal complex, meiosis, HORMAD1, RPA, RNA polymerase II, H3K27me3

## Abstract

Among eukaryotes, there are many examples of partial genome elimination during ontogenesis. A striking example of this phenomenon is the loss of entire avian chromosomes during meiosis, called a germline-restricted chromosome (GRC). The GRC is absent in somatic tissues but present in germ cells. It has been established that a prophase I male GRC is usually represented by a univalent surrounded by heterochromatin. In the present study, an immunocytochemical analysis of zebra finch spermatocytes was performed to focus on some details of this chromosome’s organization. For the first time, it was shown that a prophase I GRC contains the HORMAD1 protein, which participates in the formation of a full axial element. This GRC axial element has signs of a delay of core protein loading, probably owing to peculiarities of meiotic silencing of chromatin. The presence of repressive marks (H3K9me3 and H3K27me3) and the lack of RNA polymerase II, typically associated with active transcription, indicate transcriptional inactivation in the GRC body, despite the known activity of some genes of the GRC. Nevertheless, RPA and RAD51 proteins were found at some GRC sites, indicating the formation and repair of double-strand breaks on this chromosome. Our results provide new insights into the meiotic behavior and structure of a GRC.

## Simple Summary

In most animals, the genome does not undergo radical changes during ontogenesis. However, in some species, a programmed loss of a portion of the genome has been identified, such as the elimination of entire chromosomes during meiosis in passerine birds; this chromosome is termed the germline-restricted chromosome (GRC). The discovery of the GRC in 1998 in the zebra finch opened new avenues for understanding the phenomenon of DNA elimination. It is now known that the GRC is predominantly maternally inherited, as this chromosome is lost in males at the end of meiosis. In spermatocytes, the GRC univalent forms a distinct chromatin domain at the nuclear periphery. In the present study, immunocytochemistry showed that the components of the proteinaceous scaffold of the prophase I GRC and other chromosomes most likely load asynchronously. This is possibly due to unique aspects of chromatin conformation and transcriptional silencing in the GRC domain, where repressive chromatin marks are present, while transcriptional markers are absent. Nonetheless, some studies indicate gene expression in the GRC of several species. In this study, the molecular machinery of meiotic repair and recombination was found to be functional, as RPA and RAD51 proteins (involved in double-strand break processing) were detected at certain GRC sites. Notably, some RPA foci in the GRC univalent showed telomeric localization. It is in these chromosomal regions that female GRC homologs recombine. The observed meiotic phenomena associated with the GRC make this chromosome unique and a target for further research.

## 1. Introduction

Many eukaryotic organisms, from unicellular organisms to vertebrates, experience the loss of part of their genome during ontogenesis [1,2,3,4,5,6,7,8,9]. The different terms used to describe this phenomenon reflect the long history of its investigation. Such terms include “chromatin diminution” [10,11,12], “programmed loss of eukaryotic DNA” [13], “programmed elimination of specific DNA sequences” [14], “germ line-restricted DNA” [15], “programmed DNA deletions” [16], “programmed site-specific DNA rearrangement” [17], “programmed elimination of chromosomes” [18], and others. The term “programmed DNA elimination” is now most commonly used [19,20]. A special case of this phenomenon is the loss of entire chromosomes during avian meiosis. Such chromosomes have been named germline-restricted chromosomes (GRCs) [21].

Since their discovery in 1998 [21], GRCs have been the subject of active research, especially in the last decade [22,23,24,25]. It is now known that a GRC is probably present in all passerine birds [24,26]. In males, a GRC is usually represented by a single chromosome that has no homolog in meiocytes, while in females, two GRCs form a bivalent [27].

In spermatocytes, a GRC appears as a univalent. Such an asynaptic element can provoke a pachytene checkpoint that blocks further meiotic progression, as noted in some mammals [28]; however, the arrest of GRC-containing meiocytes has not yet been documented. During meiotic prophase I, the male GRC is typically heterochromatic and shifts to the periphery of the nucleus, generating a special body [18,22,29], very similar to the sex body described earlier [30]. Unlike the sex body, the GRC body is already formed during prophase I [18,29]. Cohesins and the SYCP3 protein are the main components of the GRC axis [18,27]. It is worth noting that the GRC contains Rad51 and MLH1 dots, which may indicate participation in repair and recombination processes [29]. Thus, the male GRC is a cohesin-containing SYCP3-positive (often fragmented) axial element (AE), which is most likely in an inactive state throughout prophase I.

The present study complements and extends existing knowledge about the structural and behavioral features of the GRC in male zebra finches, *Taeniopygia guttata* (Vieillot, 1817) (Estrildidae, Passeriformes, Aves). The zebra finch GRC is the longest acrocentric macrochromosome in the entire chromosome set (2n = 80) [27]. Drawing on prior research, we hypothesize that the male zebra finch GRC in prophase I constitutes an axial element (AE), assembled from a typical set of structural proteins, participating in repair processes, and maintaining transcriptional silence in its chromatin. To test this hypothesis, we analyzed for the first time the protein HORMAD1 as part of the multiprotein core (scaffold) of the AE, the repair protein RPA, RNA polymerase II as an indicator of transcriptional activity, and additional proteins (H3K27me3 and H3S28ph) that may contribute to chromatin inactivation. Results reveal that the prophase I male GRC possesses the typical protein profile of an AE but shows delayed structural protein loading, limited repair, and has transcriptionally inactive, condensed chromatin.

## 2. Materials and Methods

The material for the study was two male adult zebra finches *T. guttata* from a commercial stock. A total of 817 meiotic cells were examined (343 from one male, and 474 from the other). Synaptonemal complex (SC) spreads from spermatocytes were prepared according to previously described method [31] with some modifications [32]. Some details are reported by Gil-Fernández et al. [33].

Immunostaining was performed using an anti-SYCP3 (synaptonemal complex protein 3) rabbit polyclonal antibody (Abcam, Cambridge, UK, ab15093), rabbit polyclonal anti-HORMAD1 antibody (Proteintech, Rosemont, IL, USA, 13917-1-AP), rabbit polyclonal anti-RPA32/RPA2 antibody (RPA; Abcam, ab10359), mouse polyclonal anti-RAD51 antibody (Abcam, ab88572), rabbit polyclonal anti-histone H3 antibody (tri methyl K27) (H3K27me3; Abcam, ab195477), mouse monoclonal anti-RNA polymerase II antibody (RNAP II; Abcam, ab5408), mouse monoclonal anti-gammaH2A.X antibody (phospho S139) [9F3] (γH2AFX; Abcam, ab26350), mouse monoclonal anti-SUMO-1 antibody (Zymed, San Francisco, CA, USA #33-2400), mouse monoclonal anti-ubiquityl histone H2A antibody (ubiH2A; Millipore, Billerica, MA, USA, #05-678), rabbit polyclonal anti-H3K9me3 antibody (Abcam, ab8898), rat monoclonal anti-histone H3 antibody (phospho S28) (H3S28ph; Abcam, ab10543), and a human anti-centromere antibody (CREST [calcinosis, Raynaud phenomenon, esophageal dysmotility, sclerodactyly, and telangiectasia]; #90C-CS1058, Fitzgerald Industries International, Concord, MA, USA).

According to the procedure of Matveevsky et al. [34,35], immunostaining was performed. These articles provide details on the use of various primary antibodies in the first and second rounds of immunostaining. Briefly, first, the primary antibodies were added dropwise onto slides with spreads. The slides were kept in a humid chamber at 4 °C overnight. After washing in PBS three times for 2–3 min, corresponding secondary antibodies were added dropwise onto these slides. The slides were incubated in a humid chamber for 4–6 h at 37 °C. Then, after washing in PBS three times for 2–3 min, the slides were dried and mounted in Vectashield with DAPI (Vector Laboratories, Newark, NJ, USA). The slides were examined under an Axio Imager D1 microscope (Carl Zeiss, Jena, Germany) equipped with an Axiocam HRm CCD camera (Carl Zeiss). Next, if necessary, other primary antibodies were placed dropwise onto the same slides, and the immunostaining cycle was repeated. Images were processed in Adobe Photoshop CS5 Extended.

## 3. Results

At the outset, it is important to outline the main events of meiotic prophase I in most animals. SC formation is started by the core protein assembly (cohesins, SYCP3 and SYCP2, and HORMA domain-containing proteins) creating linear structures, i.e., AEs, along each chromosome during leptotene. AEs of two homologous chromosomes come closely together, align, and then synapse through a link between transverse filaments (TFs) at zygotene. At pachytene, when TFs have formed along the entire length of the homologous chromosomes, synapsis is complete. At this stage, the two AEs are called lateral elements (LEs), which are components of the mature SC. In diplotene, desynapsis of homologs occurs, and desynaptic axes undergo disassembly of core proteins. In the present study, the dynamics of various proteins of AEs/LEs and chromatin, including a GRC body, were investigated during zebra finch prophase I.

### 3.1. The Distribution of HORMA Domain-Containing Protein 1 (HORMAD1)

**Figure 1:**
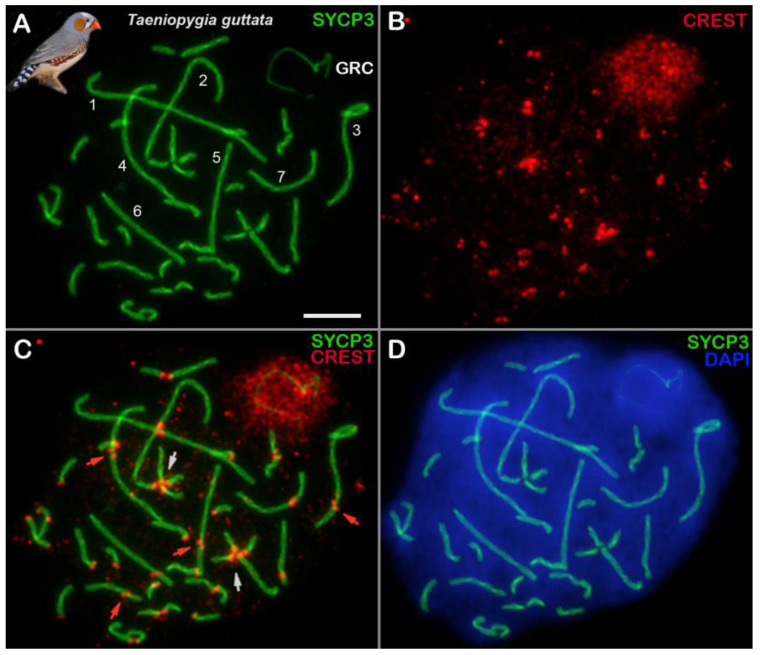
A zebra finch pachytene spermatocyte (**A**–**D**). Locations of proteins SYCP3 (green) and CREST (red) are shown. Chromatin is stained with DAPI (blue). GRC: germline-restricted chromosome. Bivalents formed by seven macrochromosomes are marked with white numbers (1–7) (**A**). White arrows indicate associations of microchromosomes via centromeric regions (**C**). Red arrows point to misalignment of centromeres in some bivalents (unaligned centromeres) (**C**). Anti-centromeric antibodies (anti-CREST) stain the chromatin of the GRC body (**B**,**C**), which re-located to the periphery of the nucleus (**D**). Scale bar = 5 µm.

In zebra finch spermatocytes, the seven SC bivalents that were formed by the macrochromosomes were clearly visible, as were the remaining SC bivalents that arose from microchromosomes and a GRC body that moved to the periphery of the nucleus (Figure 1, Figures S1 and S2A–L).

HORMAD1 is involved in SC formation and meiotic recombination [36]. In the present study, HORMAD1 was identified using the rabbit polyclonal antibody to human HORMAD1. Identifying one of the components of the proteinaceous scaffold of the GRC AE is crucial for understanding the structural integrity and full assembly of the meiotic axis.

**Figure 2:**
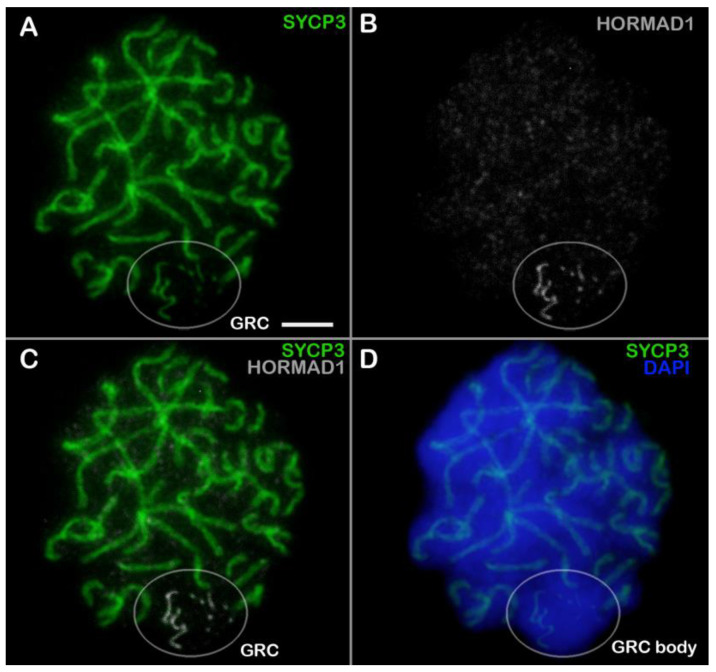
HORMAD1 localization in a zebra finch pachytene spermatocyte (**A**–**D**). Locations of proteins SYCP3 (green) and HORMAD1 (white) are shown. Chromatin is stained with DAPI (blue). GRC: germline-restricted chromosome. The white circles point to GRCs. Scale bar = 5 µm.

In leptotene, HORMAD1 appeared along short fragments of SYCP3 axes (Figure S1A–D). Within the GRC domain, HORMAD1 localization was the same as in the rest of the nucleus (Figure S1A–D). In zygotene, thin long SYCP3 axes correspond to asynaptic segments of chromosomes (Figures S1E–H and S3A–D). The GRC created a distinct domain at the nuclear periphery (Figure S1E). The GRC was visualized as SYCP3 fragments and HORMAD1-positive dots (Figure S1F–H). In early pachytene, HORMAD1 was localized only to the remaining asynaptic regions (Figure S1I–L). The GRC looked like a discontinuous SYCP3 axis, and SYCP3 and HORMAD1 signals were colocalized (Figures S1I–L and S3A–D). In mid pachytene, HORMAD1 was situated only within the GRC axis (Figure 2 and Figure S1M–P). In diplotene, HORMAD1 was detectable in desynaptic segments of chromosomes (Figure S1Q–T).

### 3.2. Distribution of a Single-Stranded DNA (ssDNA)-Binding Protein, RPA

Replication protein A (RPA) is a heterotrimeric ssDNA-binding protein complex consisting of subunits RPA70, RPA32, and RPA14 and participates in repair and recombination in eukaryotes [37]. In the present work, RPA was identified by means of the rabbit polyclonal antibody to the human replication protein A 32 kDa subunit (200 amino acid residues from the C terminus; RPA2 or RPA32) to determine its involvement in the repair process.

**Figure 3:**
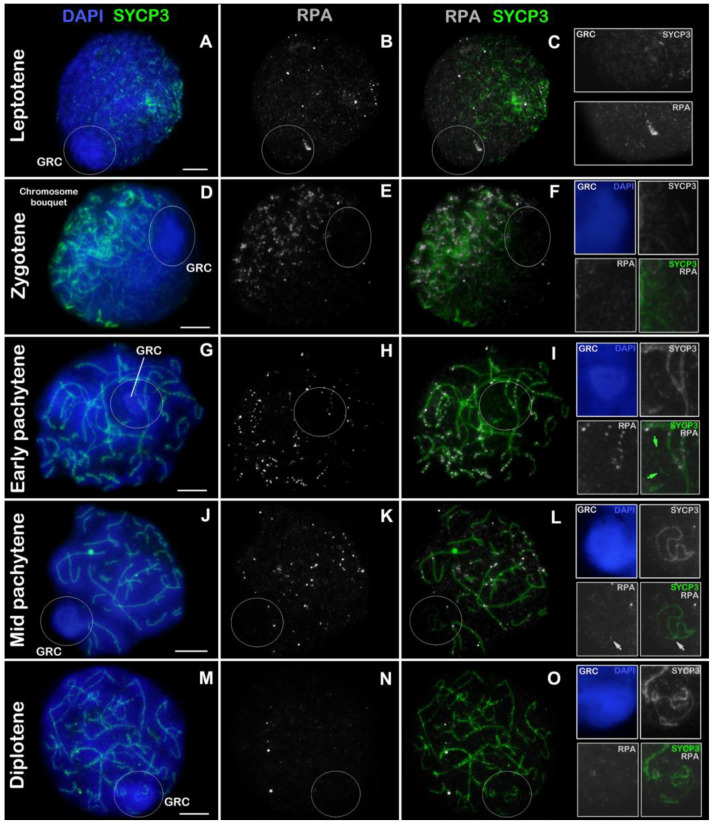
RPA distribution in a zebra finch prophase I spermatocytes. Locations of proteins SYCP3 (green) and RPA (white) are presented. Chromatin is stained with DAPI (blue). GRC: germline-restricted chromosome. (**A**–**C**) Leptotene. (**D**–**F**) Zygotene. (**G**–**I**) Early pachytene. (**J**–**L**) Mid pachytene. (**M**–**O**) Diplotene. The white circles point to GRCs. Green arrows point to SYCP3 fragments of the GRC (insets in panel (**I**)). White arrows indicate RPA dots in the GRC (insets in panel (**L**)). Scale bar = 5 µm.

In leptotene, when the SYCP3 protein was synthesized and short fragments of AEs began to emerge, RPA was detected as numerous small dim dots throughout the nucleus (Figure 3A–C). The GRC turned out to be shifted to the nuclear periphery and had a more intense DAPI signal (Figure 3A). Weak SYCP3 dots were visualized within the GRC domain, and RPA dots were positioned in the same way as in the rest of the nucleus (insets in Figure 3C).

In zygotene, chromosomes began to synapse at one of the poles of the nucleus, there-by forming the chromosome bouquet (Figure 3D). Only in the synaptic regions were clearcut specific RPA punctate signals detectable (Figure 3E,F). Within the GRC body, short SYCP3 axes were noticeable, and there were no RPA foci (insets in Figure 3F).

In early pachytene, microchromosomes were completely synapsed, whereas macro-chromosomes contained asynaptic regions (Figure 3G). RPA foci were regularly identified at synaptic sites, whereas the number of RPA dots was much lower in asynaptic axes (Figure 3H,I). The GRC was represented by short SYCP3 fragments within which there were no RPA signals (insets in Figure 3I).

In mid pachytene, all chromosomes were found to be synapsed except for the GRC (Figure 3J). The number of RPA foci was lower as compared to previous stages. One to three RPA dots were detectable within SC bivalents (Figure 3K,L). The GRC was a continuous or discontinuous SYCP3 axis twisted into a ring, figure eight, or another curved pattern within the GRC body at the periphery of the nucleus (Figure 3J,L [insets] and Figure S4A–O). Typically, GRCs had one or two (rarely three) RPA dots (insets in Figure 3L and Figure S4D–F,G–L). These dots were small and less intense as compared to RPA foci on other chromosomes. Moreover, weak RPA dots were often located at the ends of GRCs (Figure S4D–I,M–O). Sometimes, RPA dots were absent from GRCs (Figure S4A–C). Among 54 mid pachytene spermatocytes analyzed, RPA signals were absent in 19 GRCs (35.2%), present as a single dot in 24 GRCs (44.4%), and observed as two or more dots in 11 GRCs (20.4%), with 4 (7.4%) of these showing RPA localization at telomeric regions.

In diplotene, chromosomes were desynaptic, and specific RPA signals were absent, including in GRCs (Figure 3M–O, insets).

### 3.3. Distribution of RNA Polymerase II (RNAP II)

It is known that the phosphorylation of the C-terminal domain of RNAP II regulates and coordinates transcription and chromatin remodeling and modifications [38]. In this study, we used the mouse monoclonal antibody to the RNAP II C-terminal domain phosphorylated at serine 5 in tandem repeated heptapeptides YSPTSPS.

**Figure 4:**
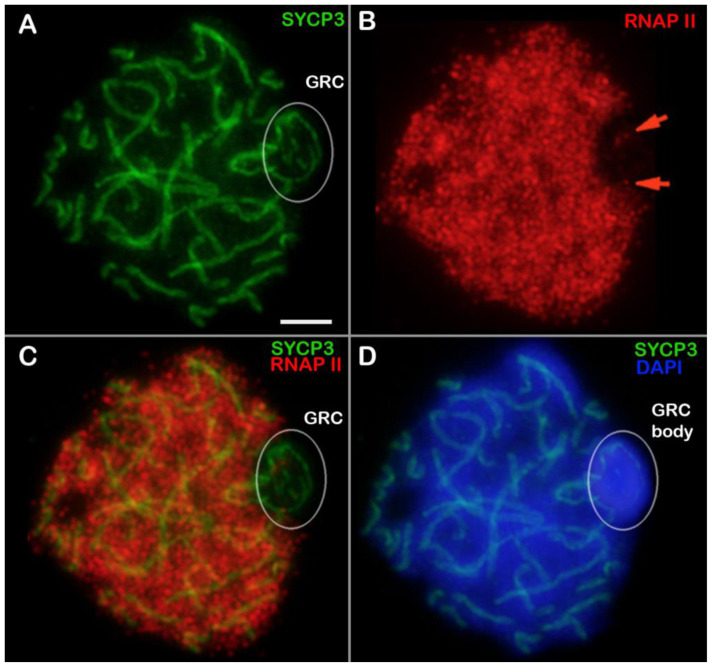
RNA polymerase II (RNAP II) localization in a zebra finch pachytene spermatocyte (**A**–**D**). Locations of SYCP3 (green) and RNAP II (red) are pointed out. Chromatin is stained with DAPI (blue). GRC: germline-restricted chromosome. The white circles point to GRCs. Red arrows point to weak RNAP II dots in the GRC axis (**B**). Scale bar = 5 µm.

The RNAP II signal filled the nuclei at all stages of prophase I (Figures S2A–L and S3A–F). In leptotene, most of the nucleus proved to be filled with RNAP II foci except for the GRC domain (Figure S2A–C). In zygotene, RNAP II signals were located independently of synaptic and asynaptic regions (Figure S2D–F). GRC body chromatin lacked RNAP II signals (Figure S2E–F), although several weak RNAP II dots were detected within the GRC axis (insets in Figure S2F). In pachytene, GRC body chromatin was RNAP II negative (Figure 4A–D and Figure S2G–I), although several RNAP II foci were also visible within the GRC axis (Figure 4B, Figures S2D–F and S5). RNAPII foci were often observed within the GRC, although they were not present in every cell (Figure S3E,F). Overall, the distribution of RNAP II was similar between zygotene and pachytene, although differences in signal intensity were noted in a few cases (Figure S5). During diplotene, the RNAP II signal exhibited a similar localization to that observed in zygotene and pachytene (Figure S2J–L). Thus, RNAP II localization in zebra finch spermatocytes is not associated with chromosomal synapsis, and GRC domain chromatin lacks RNAP II despite several dots along the GRC axis.

### 3.4. Distributions of Histones H3K27me3 and H3S28ph

Trimethylated lysine 27 on histone H3 (H3K27me3) is a silencing hallmark implicated in transcriptional inhibition within facultative heterochromatin [39,40]. In this work, we utilized the rabbit polyclonal antibody to human histone H3 trimethyl K27 (H3K27me3).

**Figure 5:**
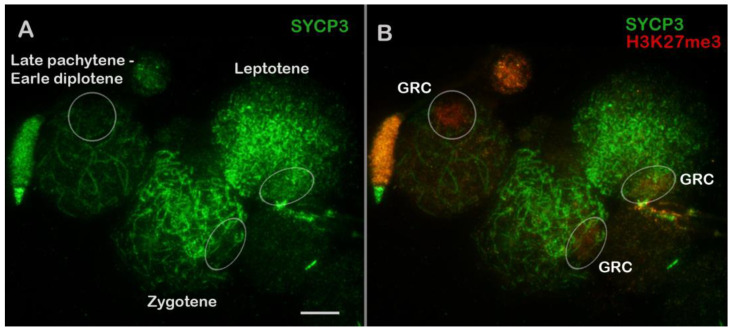
The H3K27me3 distribution in zebra finch prophase I spermatocytes (**A**,**B**). Locations of SYCP3 (green) and H3K27me3 (red) are shown. GRC: germline-restricted chromosome. The white circles point to GRCs. These data are presented more fully in Figure S6. Scale bar = 5 µm.

At all stages of prophase I, a weak H3K27me3 signal was noted in the chromatin outside SCs (Figures S6 and S7A–L). A brighter but not very dense H3K27me3 signal was observed in the chromatin of the GRC body (Figure 5, Figures S6A–F and S7C,F,I). No differences in the distribution of H3K27me3 among stages were found.

Phosphorylation of histone H3 at serine 28 (H3S28ph) is reported to be involved in transcriptional activation in somatic cells [41,42]. For instance, H3S28ph participates in the inhibition of H3K27me3 formation, thereby allowing for the accumulation of H3K27 acetylation [42,43]. It was important for us to investigate the localization of H3S28ph because transcription reactivation occurs in prophase I of some vertebrates. Here, we used the rat monoclonal antibody against H3 phosphorylated at serine 28 (H3S28ph). The entire volume of the nucleus at all stages of prophase I proved to be filled with a very weak (slightly above background) H3S28ph signal, including in the GRC domain (Figure S8A–F). Only a few round DAPI-negative structures were found to be labeled with a weak H3S28ph signal (Figure S8C,F).

### 3.5. Distributions of Some Previously Studied Proteins of Repair and Meiotic Silencing

Here, we present results of the analysis of proteins that have been studied in the zebra finch by others [18,29].

γH2AFX. In leptotene, γH2AFX foci of various intensities were located in the regions of accumulation of SYCP3 dots and short fragments of SYCP3 axes (Figure S9A–D). In zygotene, brighter γH2AFX signals were observed at the pole of the nucleus where chromosomes began to synapse (chromosomal bouquet) (Figure S9E–H). Weak γH2AFX dots were seen within the GRC body (Figure S9F–H). In pachytene, the number of γH2AFX foci was smaller than that in the preceding stage (Figure S9I–L). In the GRC domain, 2–3 γH2AFX foci were not associated with the GRC univalent (Figure S9J–L).

RAD51. In pachytene spermatocytes, 1–3 RAD51 dots were registered within the GRC domain (Figure S10). RAD51 dots were generally located near (or partially overlapped with) the GRC axis (Figure S10), similarly to another report [29]. Nonspecific RAD51 signals were observed in the chromatin of the meiotic nucleus (between SCs) (Figure S10).

SUMO-1. Although the SUMO-1 distribution was described for all stages of prophase I in another work, it was visualized only at pachytene [29]. Here, all prophase stages were visualized (Figure S11A–L). In leptotene, numerous SYCP-3 dots and fragments were present in the GRC domain. SUMO-1 was located throughout the GRC body (Figure S11A–C). In zygotene, the SUMO-1 signal was distributed in chromatin along the GRC axis (Figure S11D–F). In pachytene and diplotene, GRC body chromatin was also SUMO-1 positive (Figure S11G–L).

ubiH2A. ubiH2A here is the same as H2AK119 in ref. [29]. Nuclei at different prophase I stages manifested the same pattern: ubiH2A dots of various intensities were found to be randomly distributed throughout the nucleus, including the GRC body (Figure S12). As in the article by Schoenmakers et al. [29], our data confirmed that the specific signal is probably absent in spermatocytes.

H3K9me3. In other studies [18,29], not all stages of prophase I have been visualized in assays of H3K9me3 with in zebra finch spermatocytes. Here, we performed immunostaining for proteins SYCP3, CREST, and H3K9me3. At leptotene and zygotene, GRC domain chromatin had a weak “centromeric” signal and an intense H3K9me3 signal (Figure S13A–F). At pachytene, the CREST signal was most intense within the GRC body (Figure S13G–H). At this stage, the H3K9me3 signal in the GRC was the most intense and densest (Figure S13G–I). At diplotene, the GRC body chromatin was CREST positive and H3K9me3 positive too (Figure S13J–L). It is important to note that no H3K9me3 signal was detected in the centromere regions of standard chromosomes (Figure S13A–L).

Centromeric marker—CREST. Another noteworthy observation was that, in some spermatocytes, the chromosomes were connected through their centromeric regions (centromeric associations of chromosomes; see Section 4.4 for further details) (Figure 1 and Figure S15).

## 4. Discussion

Zebra finch pachytene karyotype includes 40 SC bivalents, with one additional meiotic element (n = 40 + 1 GRC univalent in males and n = 40 + 1 GRC bivalent in females) [27,44]. This extra element, i.e., the male GRC, became the main point of interest. Findings from this research indicate that the male GRC shows delayed incorporation of structural proteins into the AE, participates in repair processes without apparent recombination, and maintains transcriptional silence, despite chromatin activity of the rest of the nucleus during prophase I. Subsequent sections of the Section 4 will address these findings in detail.

### 4.1. The Scaffold of GRCs: Proteins Giving Rise to the AE

Meiotic chromosomes undergo morphogenesis by assembling different proteins into AEs. In mammals, the 30 to 50 nm wide core (scaffold) of the AE (axis) is composed of three protein groups: (1) major protein components (SYCP3 and SYCP2), (2) meiotic cohesins (SMC3, SMC1β, Rec8, RAD21L, and STAG3), and (3) HORMA-domain proteins (HORMAD1 and HORMAD2) [45,46]. Each of these proteins is believed to be critically important for further meiotic progression [47,48,49,50]. Cohesins play a critical role in the initial stages of AE organization [51,52,53]. Loading of cohesins Rec8 and RAD21L onto chromatin provides a framework for subsequent recruitment of other proteins that constitute the AEs [54]. Apparently, cohesin-mediated processes of loading of HORMAD1 or HORMAD2 and of SYCP3 or SYCP2 occur in parallel and independently of each other, although after the incorporation, these protein components interact with each other, stabilizing the AE’s scaffold and the SCs [55]. Thus, now there is an approximate understanding of the key steps of AE formation. This section addresses this issue in relation to the zebra finch GRC.

**Figure 6:**
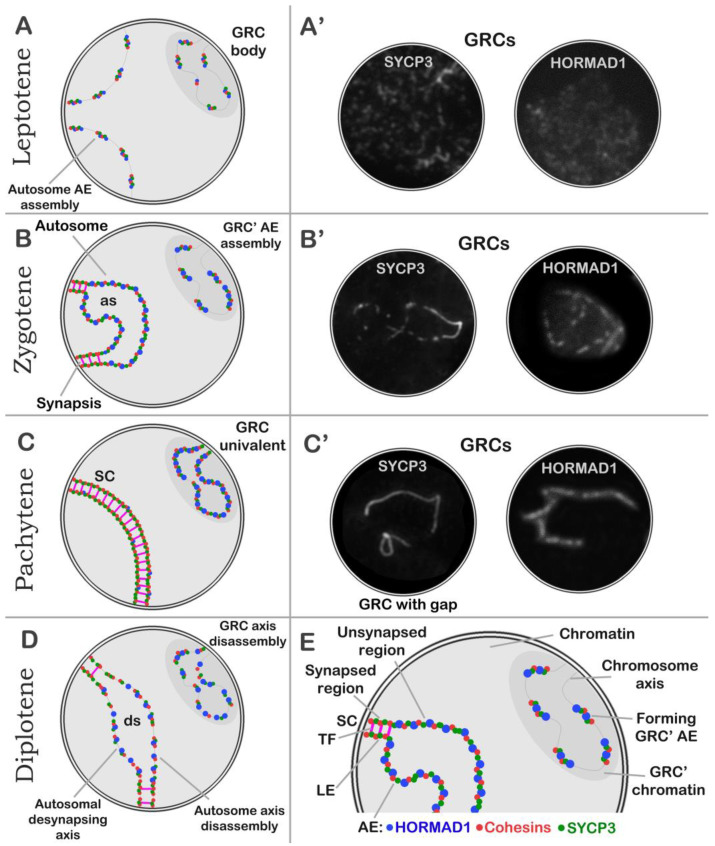
Dynamics of GRC structure at prophase I of zebra finch meiosis. Schematic representations of the structure and behavior of meiotic chromosomes are depicted. As the scheme is simplified, it does not capture the chromatin’s loop organization. Abbreviations: GRC, germline-restricted chromosome; SC, synaptonemal complex; AE, axial element; LE, lateral element of SC; TF, transverse filament of central space of SC; as, asynapsis area; ds, desynapsis area. In AEs/LEs of SCs: HORMAD1 (blue dots), cohesins (red dots), and SYCP3 (green dots). Typically, the GRC was found to create a distinct chromatin domain at the nuclear periphery during prophase I (**A**–**D**). (**A**) Leptotene. At this stage, core proteins (cohesins, HORMAD1 and SYCP3) (**A′**) are loaded into the chromosome axis; therefore, the nascent proteinaceous scaffolds of the AEs of autosomes and GRC can be observed. (**B**) Zygotene. Chromosomes form a bouquet. Autosomal AEs arise. Two AEs of homologous chromosomes align with each other and begin to form SC segments. SC regions either lack HORMAD1 or contain rare stand-alone signals. Unlike autosomes, where protein assembly of AEs was completed, loading of core proteins continues into the AE of the GRC (protein loading delay) (**B′**). Asynaptic regions and some regions of the GRC univalent contain a large amount of HORMAD1. (**C**) Pachytene. Autosomes are fully synapsed. Loading of core proteins into the GRC AE is almost complete. Sometimes, some regions of the GRC remain without loaded core proteins (fragmented GRC AEs or GRC with gaps) (**C′**). Unlike autosomes, the GRC univalent has a lot of HORMAD1. (**D**) Diplotene. Chromosomes are desynapsed. The GRC and desynapsing axes of autosomes undergo protein disassembly. Desynapsing segments of autosomes and GRC are enriched with HORMAD1. (**E**) Explanatory diagram.

One protein of the cohesin complex—SMC3 (structural maintenance of chromo-somes protein 3)—is present in chromosomes of the A-set and in the GRC AE at all stages of prophase I in zebra finch spermatocytes [18,27]. SMC3 probably forms a framework of the GRC AE together with other cohesins for loading of other proteins. A similar pattern of distribution in GRC has been documented for the SYCP3 protein [27,29]. A component of the AE that has not been analyzed to date is the HORMA domain-containing proteins (HORMAD1 and HORMAD2). HORMAD1 promotes SC formation and facilitates chromosome synapsis and recombination [55,56,57,58]. It has been revealed that as SCs arise, HORMAD1 is depleted from the chromosome axes [59]. This pattern was confirmed in the present study. Namely, in zebra finch spermatocytes, asynaptic autosomal axes contained a strong HORMAD1 signal, which disappeared when the chromosomes synapsed and formed SCs. Because the extra chromosome is asynaptic, the GRC axis contained HORMAD1 throughout prophase I while colocalizing with SYCP3 (Figure 6).

Our comparison of distributions of core proteins in standard and accessory chromosomes suggests that the recruitment of these proteins into the GRC is delayed: in zygotene, when autosomal AEs have continuous SYCP3 localization, the GRC axis is often fragmented (multiple SYCP3 fragments, AE with gaps) (Figure 6B,B′ and Figure S1J–L; insets in Figure 3I and Figure S2F). In some cases, the lengths of SYCP3 and HORMAD1 fragments in the GRC at zygotene and pachytene contradict the fact that this chromosome is the longest one of the set (a macrochromosome), as demonstrated elsewhere [21,27]. There may be several explanations. One scenario implies that long regions of the chromosome axis loop out between the core protein fragments and remain without protein loading for some reason (a GRC with gaps). A possible reason is the specific conformation of chromatin within the GRC body. If we look at Figure S13, we can see a dense H3K9me3 signal (especially in pachytene), which may indicate a high level of heterochromatinization of the GRC body. These specific conditions may prevent protein loading in sometimes.

The second scenario implies that the GRC axis folds into a tangle or tangle-like shapes, resulting in a temporary delay of the incorporation of core proteins into AEs. Given that GRCs sometimes represent a continuous multiprotein axis, it can be assumed that proteins are able to finally complete the assembly of the GRC’s AEs. A third scenario could be that the GRC is a constantly changing element in evolution. As Borodin [8] notes, some genes are transferred to GRCs and amplified, while others are deleted, and these processes could affect the size of this extra chromosome even in closely related species. It is plausible that “old” and evolutionarily dynamic segments of the GRC differ in their rates of protein loading in prophase I, yet no direct data currently confirm this possibility. All scenarios may occur simultaneously or in some other variations.

A comparison of the GRC univalent with prophase I univalents in other animals may shed light on the specificity of the protein-loading delay. Perhaps the absence of a homo-log manifests itself as specific patterns of structural proteins of AEs. The most striking example of univalents is supernumerary chromosomes (B chromosomes), especially in the narrow-headed vole, in which Bs are clearly visible linear univalents [60], in contrast to the B chromosomes of foxes and Korean field mouse [61,62,63]. In our recent paper about voles, pachytene Bs’ univalents had a continuous SYCP3 axis with uneven thickening and thus were similar to Y- and X-axes [60]. In zygotene spermatocytes, however, when the thin AEs of autosomes were formed, Bs were already visually more striking univalents, usually with a continuous and clumpy distribution of SYCP3 (our unpublished data). Loading of core proteins into AEs probably proceeds differently (both structurally and temporally) if we compare the zebra finch GRC and vole Bs.

Localization of a centromere allows us to determine the chromosome type. The GRC is acrocentric (Figures S11I and S13H,K), which is in agreement with literature data [18,27,64]. It should also be noted that sometimes, the centromeric regions of meiotic chromosomes were found to be unaligned (there was no alignment) in our work when the centromeric dots in the bivalent were located separately (Figure 1, Figures S11I and S13H). These patterns have been observed both in other birds [24,26] and in various mammalian species [for example, see Figure 3 in ref. [65] or Figure 1a in ref. [66]. It is possible that parents of the zebra finches under study come from different parts of the geographic range, where minor differences in the location of centromeres in some chromosomes could exist. It is plausible that the misalignment is connected to a synaptic delay in the pericentromeric regions.

Therefore, despite specific structural variations, the prophase I GRC is a full AE consisting of a classic set of proteins, similarly to autosomal AEs in meiocytes of zebra finches and other animals.

### 4.2. GRCs and Proteins Participating in Repair and Recombination

It is known that at the beginning of meiotic prophase I in mammals, ~150–300 programmed double-strand breaks (DSBs) are generated [67]. In response to DNA damage, histone H2AX begins to be phosphorylated at serine 139 (γH2AFX) [68]. For instance, in mice at the end of leptotene and early and mid-zygotene, the entire nucleus volume is filled with an intense γH2AFX cloud [69]. The zebra finch features a different γH2AFX pattern. Consistent with previous data [29], we again showed that the distribution of γH2AFX in leptotene and pachytene is limited to some regions of the nucleus, whereas in zygotene, γH2AFX is localized to the region of the nucleus where chromosomes enter synapsis. Most likely, DSBs are induced in these chromosome regions. Only a few weak γH2AFX foci were observed within the GRC body during prophase I in our work.

Processing of DSBs results in ssDNA ends, which are initially coated by RPA to prevent degradation and secondary-structure formation [70]. RPA is subsequently displaced by RAD51 and DMC1 proteins, which promote the active homology search and carry out strand invasion [71]. In the present paper, RPA was analyzed for the first time in the zebra finch. Numerous specific RPA foci were observed at the pole of the zygotene nucleus where DSBs were found to be actively induced (and labeled with γH2AFX) (Figure 3D–F), as expected. The number of these foci decreased toward pachytene and diplotene.

Of note, several weak RPA and RAD51 foci were noticed in the GRC axis. Provided that the identified weak RPA foci are accurate and valid, it can be proposed that DSBs are generated at one, two, or three sites within the GRC. Notably, in several nuclei in the GRC axis, (near-)telomeric localization of RPA was registered but not for RAD51 [29]. Although the GRC likely does not recombine in males [no MLH1 dots in GRCs [24], DSBs are processed in those telomeric segments where recombination occurs in a female GRC bivalent [27]. As reported by Schoenmakers et al. [29], a possible obstacle to the incorporation of repair and recombination proteins into the male GRC is dense heterochromatic packing in the GRC body. Indeed, it has been demonstrated in yeast that chromatin accessibility (conformation) is one of determinants of the number of DSBs [72,73]. The absence of recombination (in males) or highly restricted recombination (in females) may have evolutionary consequences [7,8]. Thus, the male GRC represents a chromosome with limited repair.

### 4.3. Meiotic Silencing of the GRC: Proteins Forming Inactive Chromatin

The zebra finch prophase I GRC body is a chromatin domain localized typically to the nuclear periphery from leptotene to diakinesis and accumulating various chromatin modification marks (Figure S14). GRC chromatin is marked by SUMO-1 and histones H3K9me3, macroH2A, H3K27me3, H4K16ac, H3K20me3, and H3K20me2 but not by H3K4me3, H3S28ph, H3S10ph, H3K9ac, H4K5ac, H4K8ac, and ubiH2A [[18,29] and this study]. The presence of H3K9me3—a marker of constitutive heterochromatin—confirms the heterochromatic nature of the GRC body [74]. This observation is consistent with the presence of HP1, H3K20me3, and H3K20me2 in the GRC, the localization of which is associated with heterochromatic regions [75,76,77]. In our analysis, there was a weak H3K27me3 signal in GRC chromatin; this type of histone is considered a marker of facultative heterochromatin [78]. There is a point of view that H3K27me3 is able to compensate for the role of H3K9me3 in the organization of heterochromatin when histone H3K9 is demethylated [79]. It is likely that the GRC body shows some alternation of facultative and constitutive heterochromatin, and these differences may yield dissimilar conformational states for, e.g., entry of other proteins.

Our results also indicate that, the chromatin of the GRC body is characterized by the absence of transcriptional activity (i.e., is an RNAP II-negative domain), or at the very least, by substantially diminished transcriptional levels. This finding is supported by the absence of methylated H3K4 and acetylated H4 [18,29], which are related to transcriptionally active chromatin [69,80,81,82]. It is important to note, however, that in our analysis, the GRC contained several weak RNAP II dots similar to the RNAP II clusters in lampbrush GRCs of females [24], implying the presence of transcription sites. This notion is consistent with the fact that many (but not all) GRC genes are expressed across bird species [23,25,83]. It remains unclear whether the identified RNAP II foci reflect genuine gene expression in the GRC. This question was not addressed in the current investigation.

Another important observation is that zebra finch meiotic chromatin is transcriptionally active throughout prophase I (Figure S14), as demonstrated in chickens [84]. This phenomenon distinguishes birds from some animal species that show synapsis-dependent or synapsis-independent transcriptional reactivation in mid prophase I [69,85,86].

A univalent—as illegitimate asynapsis in pachytene cells—represents a target for an epigenetic response. It is believed that the asynaptic region of parts of a chromosome or asynaptic chromosome can undergo transcriptional inactivation, which is called meiotic silencing of unsynapsed chromatin (MSUC) [87,88]. If a gene critical for meiotic progression is inactivated, such a spermatocyte enters apoptosis, which has been demonstrated for some mammals [89]. Nonetheless, there is an exception. Heteromorphic sex chromosomes are synapsed in a short region, thereby preserving long asynaptic regions. In pachytene, such chromosomes move to the periphery of the nucleus and form a special sex (XY) body (in placental mammals) [30], whose chromatin is inactivated (meiotic sex chromosome inactivation, MSCI) [69,90]. Thus, asynapsis of sex chromosomes is not recognized by the monitoring of the pachytene checkpoint. A similar epigenetic strategy may have been evolutionarily adapted to the GRC (and possibly to the avian ZW). Starting from preleptotene throughout prophase I, the GRC gives rise to a separate chromatin cluster at the nuclear periphery and goes through meiotic silencing. This phenomenon is called “meiotic silencing prior to synapsis” [29]. This strategy is probably not accidental because some genes situated in the GRC may be necessary at some stages of spermatogenesis.

Consequently, the prophase I GRC forms a distinct cluster with a unique epigenetic program of chromatin changes for its transcriptional inactivation, likely to overcome meiotic checkpoints.

### 4.4. Sporadic Association of Prophase I Chromosomes via Centromeric Regions

Another chromosomal feature of zebra finch spermatocytes has not been emphasized previously. In our analysis, some chromosomes, typically microchromosomes, proved to be associated via centromeric regions. Such centromeric chromosome contacts were detected in 14 of 87 (16.1%) and 9 of 65 (13.8%) counted pachytene nuclei from the two zebra finch males (Figure 1 and Figure S15A–H). Similar centromere patterns have been visualized in spermatocytes from the zebra finch and other passerine birds earlier [in the zebra finch: see Figure S3A in [24]; in the barn swallow: see Figure S3P in [24]; and in the great tit: see Figure 3D in [91]]. In our work, regions of autosomal centromeric contacts did not contain H3K9me3-heterochromatin and therefore were most likely not related to a condensed state of the surrounding chromatin.

Moreover, in one case, contacts of centromeres of the GRC and of microchromosomes were detectable (Figure S15G). This association may involve the heterochromatic nature of the GRC body (Figure S15H). Such ectopic contacts of the GRC univalent with microchromosomes can be interpreted as signs of meiotic arrest because the involvement of chromosomes in the heterochromatic cloud of the GRC body may modify or disrupt normal expression of important genes. On the other hand, this is an extremely rare phenomenon that probably has no evolutionary significance.

It is known that satellite sequences can be involved in centromeric connections of chromosomes and interchromosomal contacts, although they are usually associated with pericentromeric heterochromatin and chromocenter formation [92]. Centromeric associations of zebra finch chromosomes were not mediated by H3K9me3 heterochromatin in our analysis. It is possible that centromeric contacts of chromosomes can be determined by other factors. The identification of centromere-specific repeats on prophase I chromosomes [93] of zebra finch may provide insights into the true nature of these associations. Indeed, centromeric sequences rapidly change evolutionarily, as, for example, in other passerine species: the common nightingale and the thrush nightingale [94]. Additional studies on meiotic centromeres of zebra finches will be worthwhile.

## 5. Conclusions

The zebra finch GRC is a special chromosome with unique behavioral patterns. Despite the absence of recombination, the male GRC is capable of limited formation and repair of DSBs at sites it recombines in females. Probably, such sites retain the ability to recombine if the homolog is present in the nucleus. Another feature is the asymmetric formation of AEs in the GRC and other chromosomes, which may be due to the heterochromatic state of the GRC body and/or the peculiarities of meiotic silencing. It is possible that the delay in protein loading into the AE reflects a morpho-functional consequence of the differential evolutionary rates of the new and old GRC regions, although this remains speculative due to the lack of direct evidence.

It is noteworthy that two strategies regulate GRC gene expression. First, temporary repression of GRC genes occurs during meiosis, after which the GRC is expelled from the cell. The first strategy—meiotic silencing—is executed via histone modifications in GRC chromatin. However, transcriptional inactivation in the GRC is not complete, as some genes on the chromosome are known to be expressed [23,25,83]. Interestingly, GRC chromatin shows signs of overall silencing, despite certain genes remaining active. This phenomenon may be due to the presence of both facultative and constitutive heterochromatin regions.

In addition to the features discovered here, the GRC has other specific characteristics. These include predominant maternal inheritance [27,95], sexual dimorphism in meiotic behavior, polymorphism, and mosaicism of this chromosome in some songbirds [26,91,96]. Moreover, GRCs of different birds—including closely related species—differ not only in size but also in genetic composition [23,24] (more details in ref. [8]). This indicates a high rate of GRC evolution as noted in some publications ([8] and refs there in). Many papers and reviews discuss the key issue: the biological significance of GRCs [[6,7,8,24] and others]. Given that this issue was not the primary focus of this study, we will postpone our thoughts on this topic until our future investigations.

Similar studies on zebra finch female meiosis, especially examining the female GRC and the ZW pair with heterologous synapsis and transcriptional inactivity, may provide further insights for comparing with male meiosis.

## Data Availability

Data are contained within the article and Supplementary Materials.

## Supplementary Materials

The following supporting information can be downloaded at: https://www.mdpi.com/article/10.3390/ani14223246/s1. Figure S1: HORMAD1 distribution in zebra finch prophase I spermatocytes; Figure S2: RNA polymerase II (RNAP II) distribution in zebra finch prophase I spermatocytes; Figure S3: HORMAD1 and RNAP II distribution in zebra finch zygotene and pachytene spermatocytes; Figure S4: RPA distribution in zebra finch GRCs at pachytene spermatocytes; Figure S5: RNAP II distribution in zebra finch zygotene and pachytene spermatocytes; Figure S6: H3K27me3 distribution in zebra finch prophase I spermatocytes; Figure S7: H3K27me3 distribution in zebra finch at different stages of meiotic prophase I; Figure S8: H3S28ph distribution in zebra finch pachytene spermatocytes; Figure S9: γH2AFX distribution in zebra finch prophase I spermatocytes; Figure S10: RAD51 distribution in zebra finch pachytene spermatocytes; Figure S11: SUMO-1 distribution in zebra finch prophase I spermatocytes; Figure S12: ubiH2A distribution in zebra finch prophase I spermatocytes; Figure S13: H3K9me3 distribution in zebra finch prophase I spermatocytes; Figure S14: Meiotic silencing of prophase I GRC in zebra finch meiosis; Figure S15: Centromeric association of chromosomes in zebra finch prophase I spermatocytes.
