# Supplementary material for: The Germline-Restricted Chromosome of Male Zebra Finches in Meiotic Prophase I: A Proteinaceous Scaffold and Chromatin Modifications
Source: Animals (Basel). 2024 Nov 12;14(22):3246. doi: 10.3390/ani14223246 (PMC11591414; doi:10.3390/ani14223246)
Supplement: Supplementary file 1 [file animals-14-03246-s001.zip › animals-3282002-supplementary.pdf]

animals

Supplementary materials

The Germline-Restricted Chromosome of Male Zebra Finches in Meiotic Prophase I: a Proteinaceous Scaffold and Chromatin Modifications

Sergey Matveevsky [1,]*

[1] Vavilov Institute of General Genetics, Russian Academy of Sciences, 119991 Moscow, Russia
* Correspondence: sergey8585@mail.ru, s.matveevsky@vigg.ru

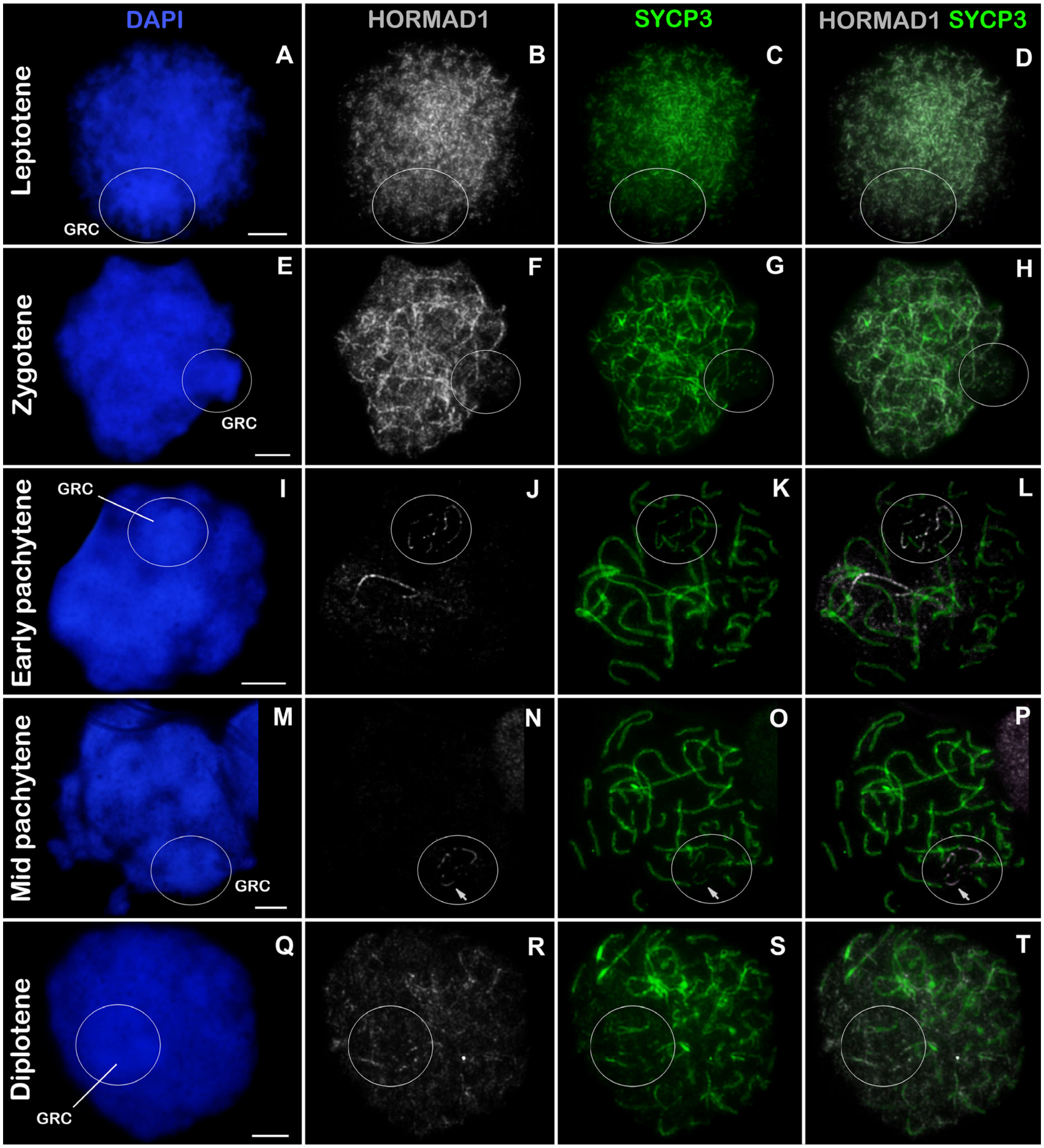

Figure S1. HORMAD1 distribution in zebra finch prophase I spermatocytes. The localizations of SYCP3 (green) and HORMAD1 (white) proteins are shown. Chromatin is stained by DAPI (blue). GRC – germline-restricted chromosome. **A-D.** Leptotene; **E-H.** Zygotene; **I-L.** Early pachytene; **M-P.** Mid pachytene; **Q-T.** Diplotene. The white circles point to GRCs. White arrow points to HORMAD1-positive fragment of the GRC (N-P). Scale bar = 5 μm.

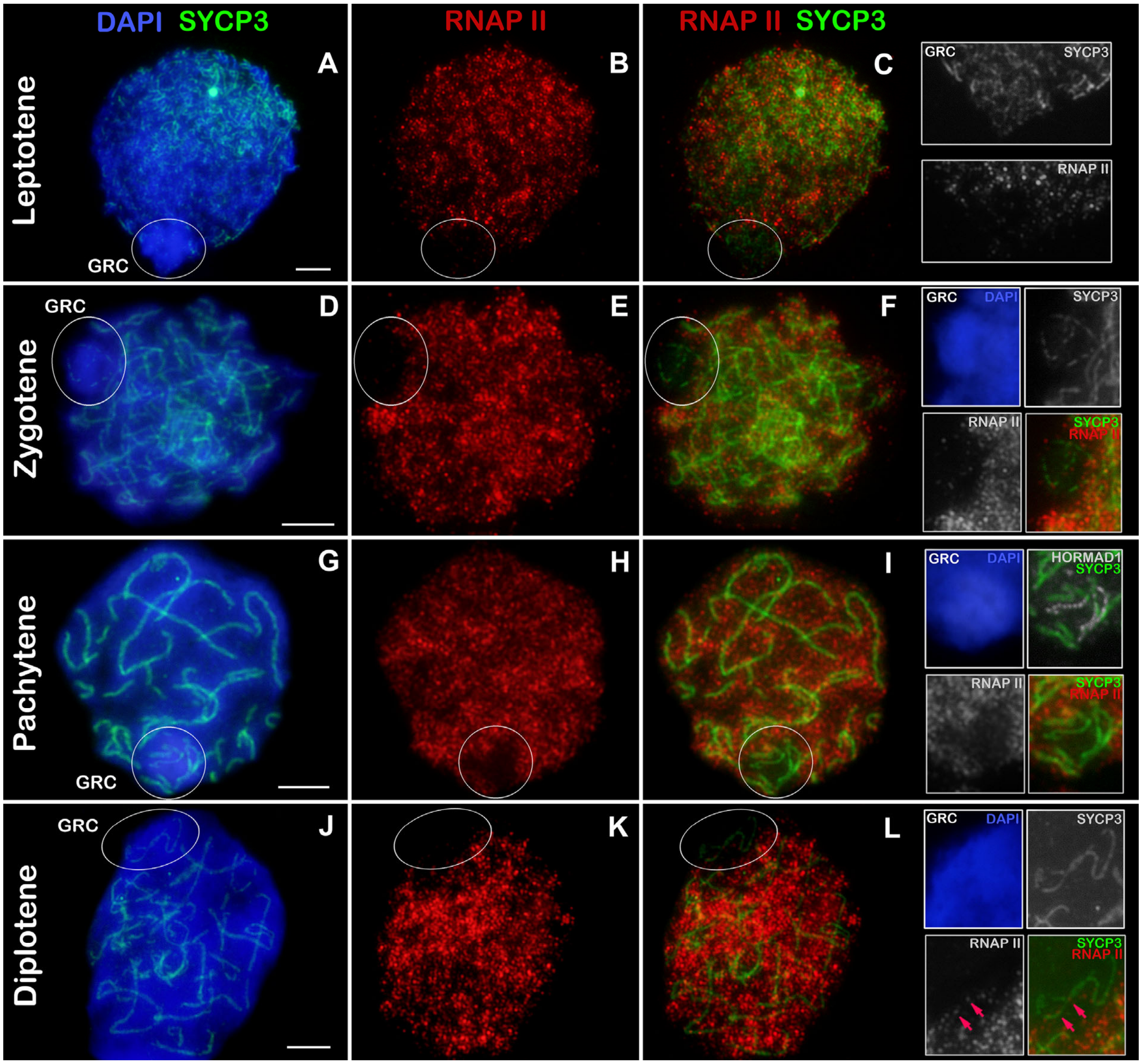

Figure S2. RNA polymerase II (RNAP II) distribution in zebra finch prophase I spermatocytes. The localizations of SYCP3 (green), RNAP II (red) and HORMAD1 (white; insets in I) are shown. Chromatin is stained by DAPI (blue). GRC – germline-restricted chromosome. **A-C.** Leptotene; **D-F.** Zygotene; **G-I.** Pachytene; **J-L.** Diplotene. The white circles point to GRCs. Magenta arrows point to weak RNAP II dots in GRC axis (insets in L). Scale bar = 5 µm.

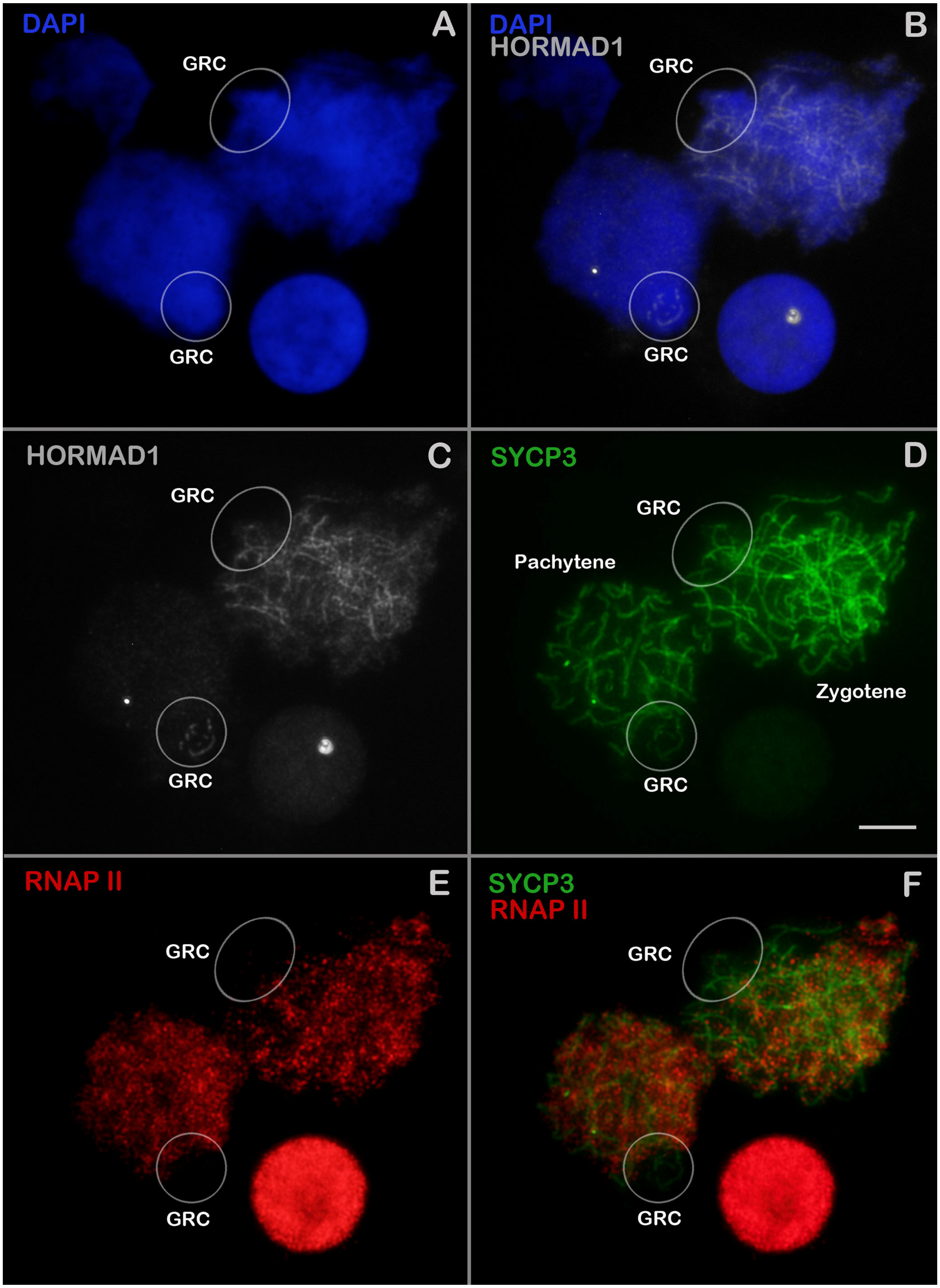

Figure S3. HORMAD1 and RNA polymerase II (RNAP II) distribution in zebra finch zygotene (right) and pachytene (left) spermatocytes (A-F). The localizations of SYCP3 (green), HORMAD1 (grey) and RNAP II (red) are shown. Chromatin is stained by DAPI (blue). GRC – germline-restricted chromosome. The white circles point to GRCs. Scale bar = 5 μm.

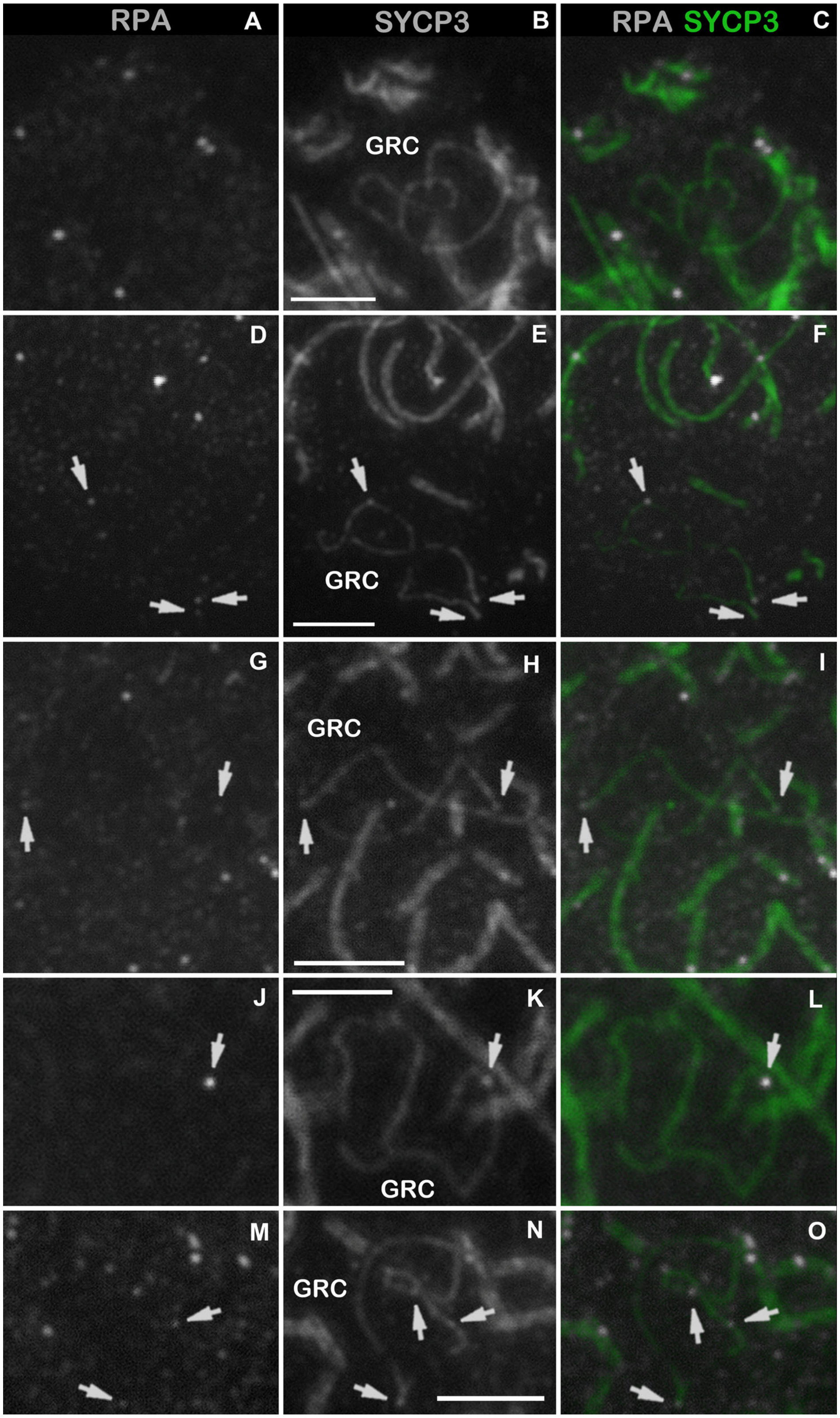

Figure S4. RPA distribution in zebra finch GRCs at pachytene spermatocytes. The localizations of SYCP3 (green) and RPA (white) proteins are shown. GRC – germline-restricted chromosome. Parts of the pachytene nuclei are represented (A-C, D-F, G-I, J-L, M-O). White arrows point to the RPA dots in GRC. Scale bar = 5 μm.

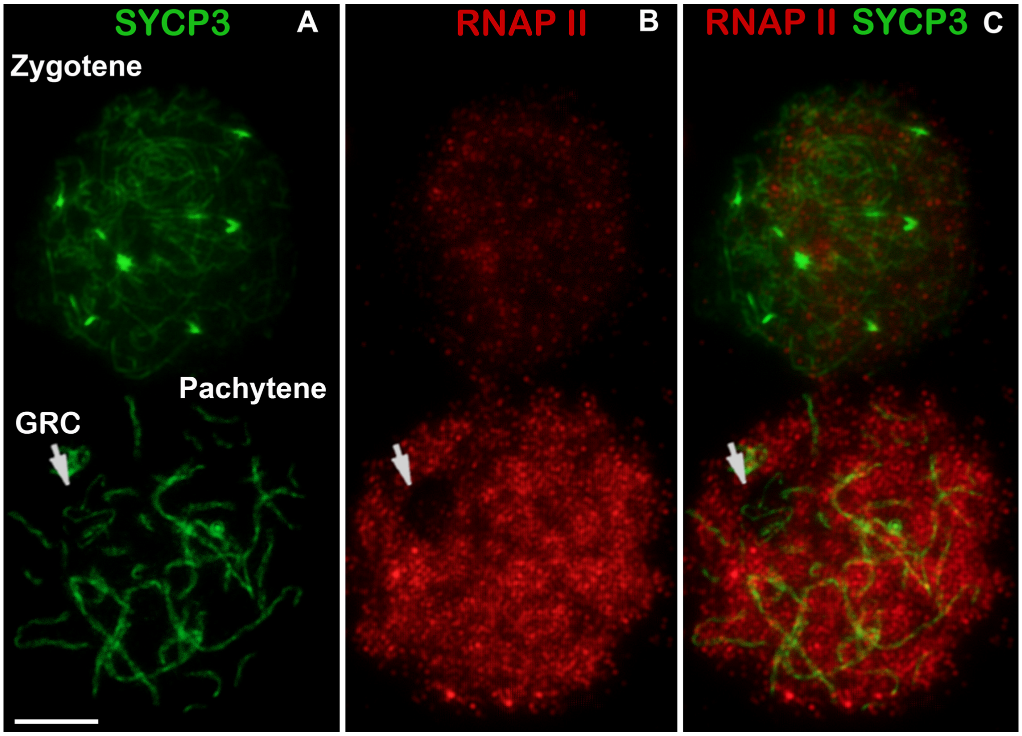

Figure S5. RNA polymerase II (RNAP II) distribution in zebra finch zygotene (top) and pachytene (bottom) spermatocytes (A-C). The localizations of SYCP3 (green) and RNAP II (red) are shown. GRC – germline-restricted chromosome. White arrows point to GRC axis. Scale bar = 5 μm.

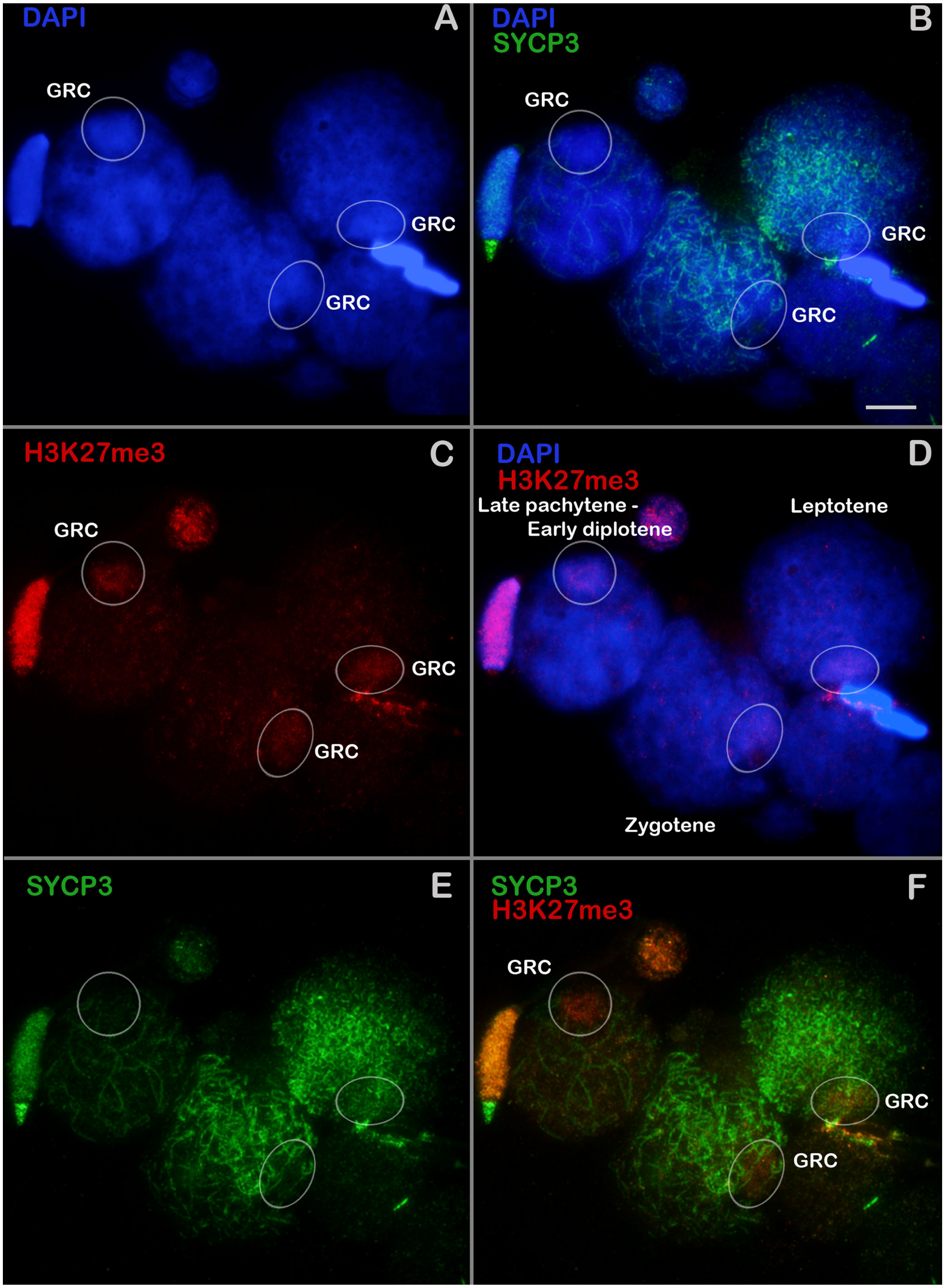

Figure S6. H3K27me3 distribution in zebra finch prophase I spermatocytes. The localizations of SYCP3 (green) and H3K27me3 (red) are shown. Chromatin is stained by DAPI (blue). GRC – germline-restricted chromosome. The white circles point to GRCs. Scale bar = 5 μm.

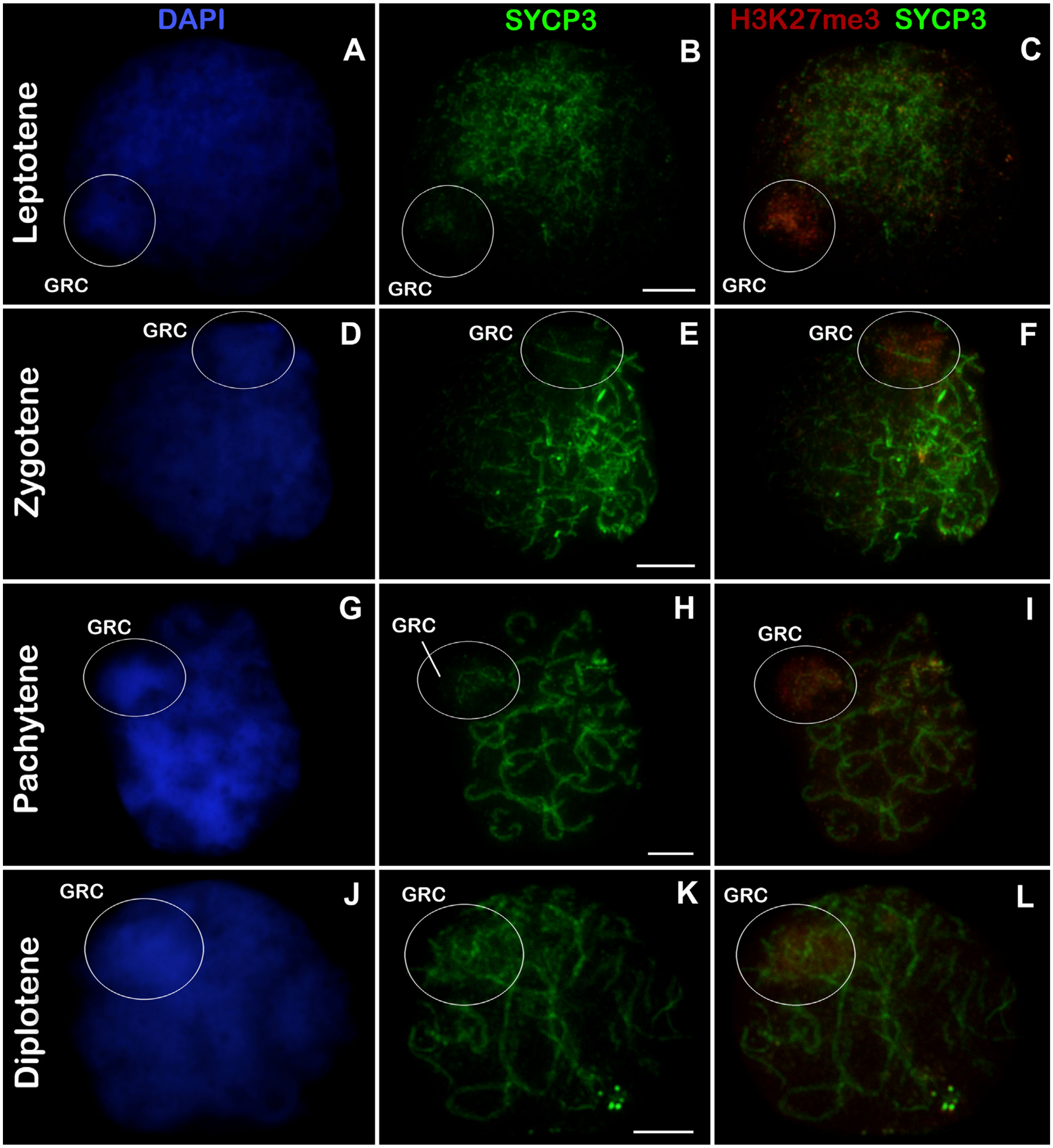

Figure S7. H3K27me3 distribution in zebra finch at different stages of meiotic prophase I. The localizations of SYCP3 (green) and H3K27me3 (red) are shown. Chromatin is stained by DAPI (blue). GRC – germline-restricted chromosome. **A-C.** Leptotene; **D-F.** Zygotene; **G-I.** Pachytene; **J-L.** Diplotene. The white circles point to GRCs. Scale bar = 5 µm.

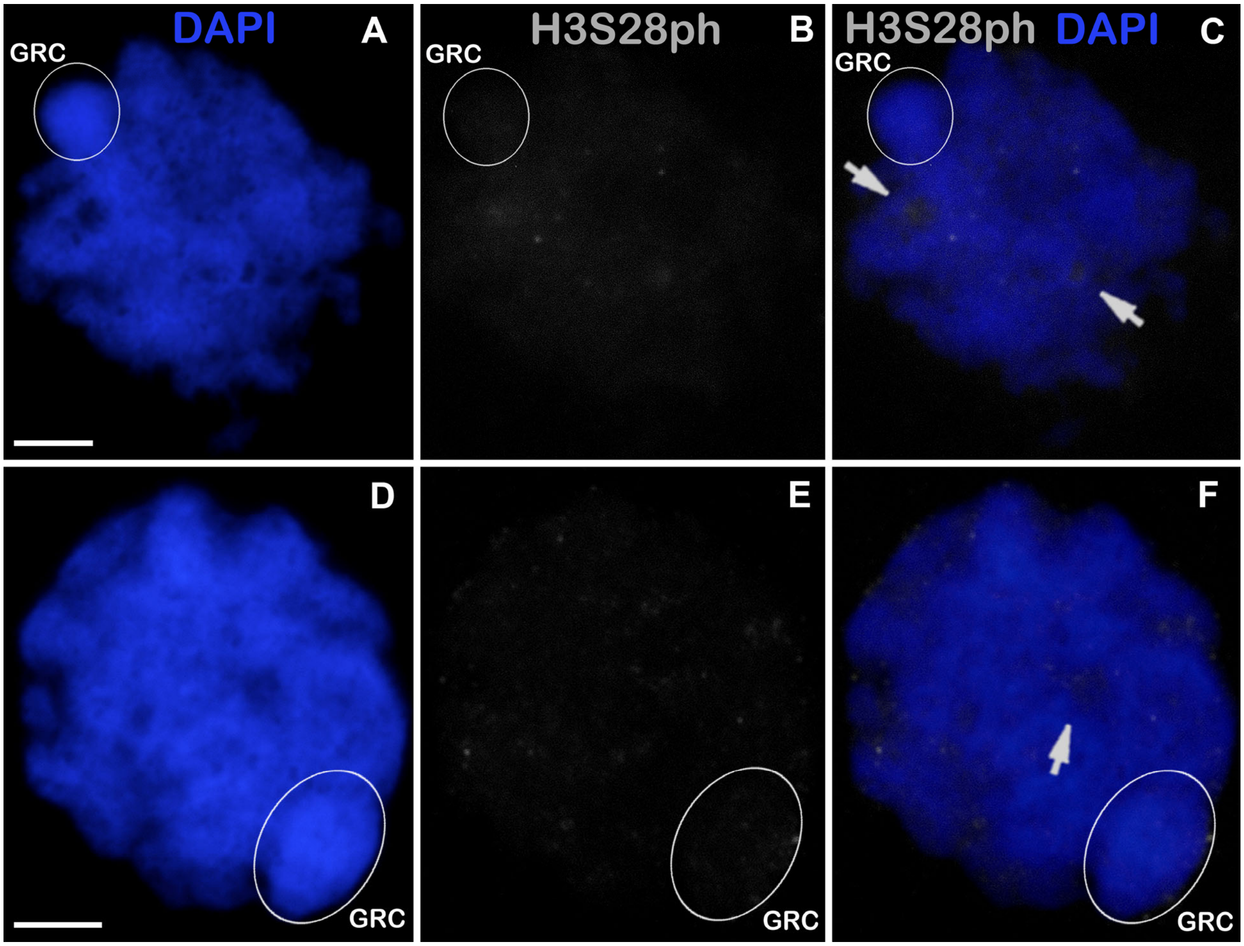

Figure S8. H3S28ph distribution in zebra finch pachytene spermatocytes (A-C, D-F). The localization of **H3S28ph** (white) is shown. Chromatin is stained by DAPI (blue). GRC – germline-restricted chromosome. The white circles point to GRCs. White arrows point to DAPI negative oval or round structures in which a slightly more intense H3S28ph signal is detected. Scale bar = 5 μm.

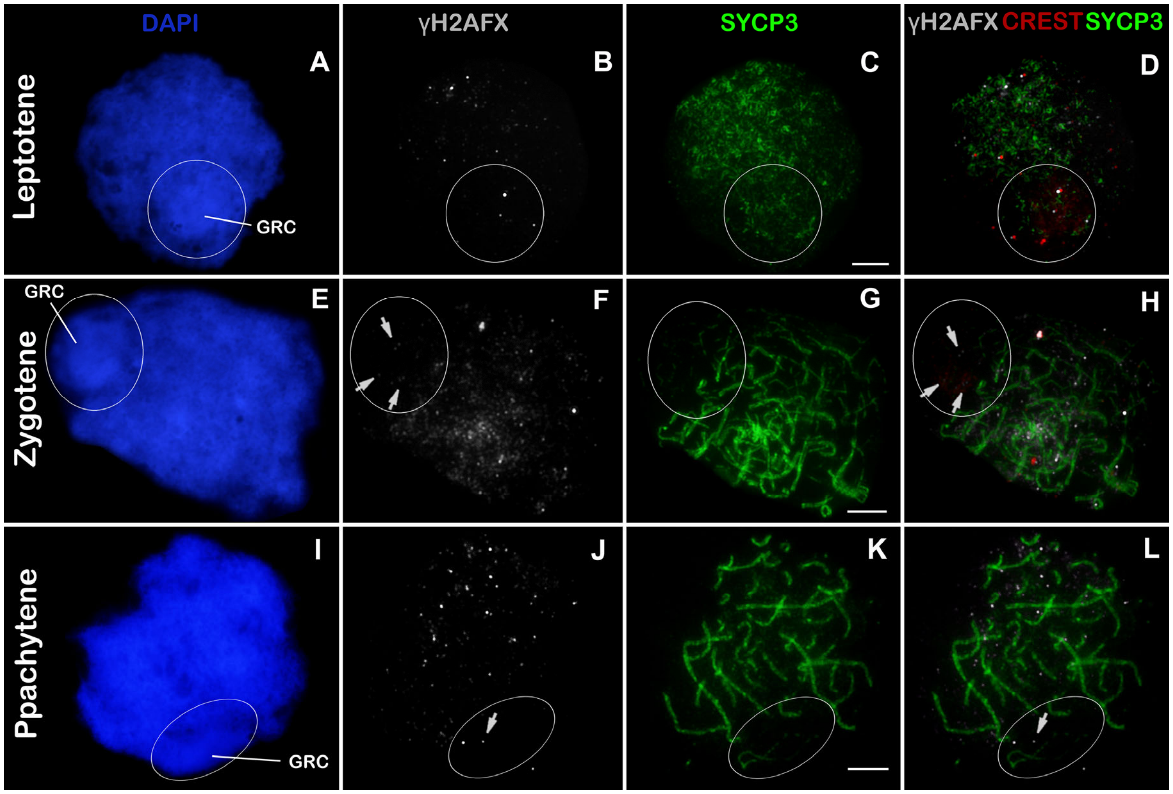

Figure S9. γH2AFX distribution in zebra finch prophase I spermatocytes. The localizations of SYCP3 (green), γH2AFX (white) and CREST (red; for D and H) are shown. Chromatin is stained by DAPI (blue). GRC – germline-restricted chromosome. White arrows point to weak γH2AFX foci in the GRC body. **A-D.** Leptotene; **E-H.** Zygotene; **I-L.** Pachytene. The white circles point to GRCs. Scale bar = 5 μm.

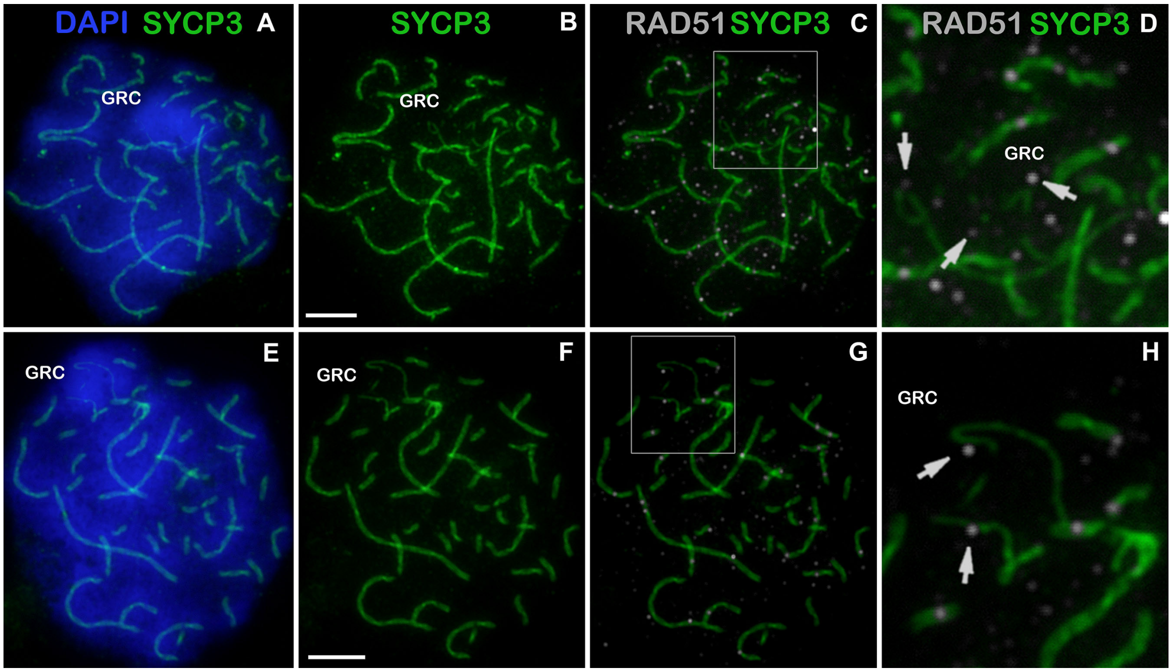

Figure S10. RAD51 distribution in zebra finch pachytene spermatocytes (A-D, E-H). The localizations of SYCP3 (green) and RAD51 (white) are shown. Chromatin is stained by DAPI (blue). GRC – germline-restricted chromosome. White arrows point to RAD51 foci. The areas of the nucleus in the gray frames (C, G) are shown in enlarged format (D, H). Scale bar = 5 μm.

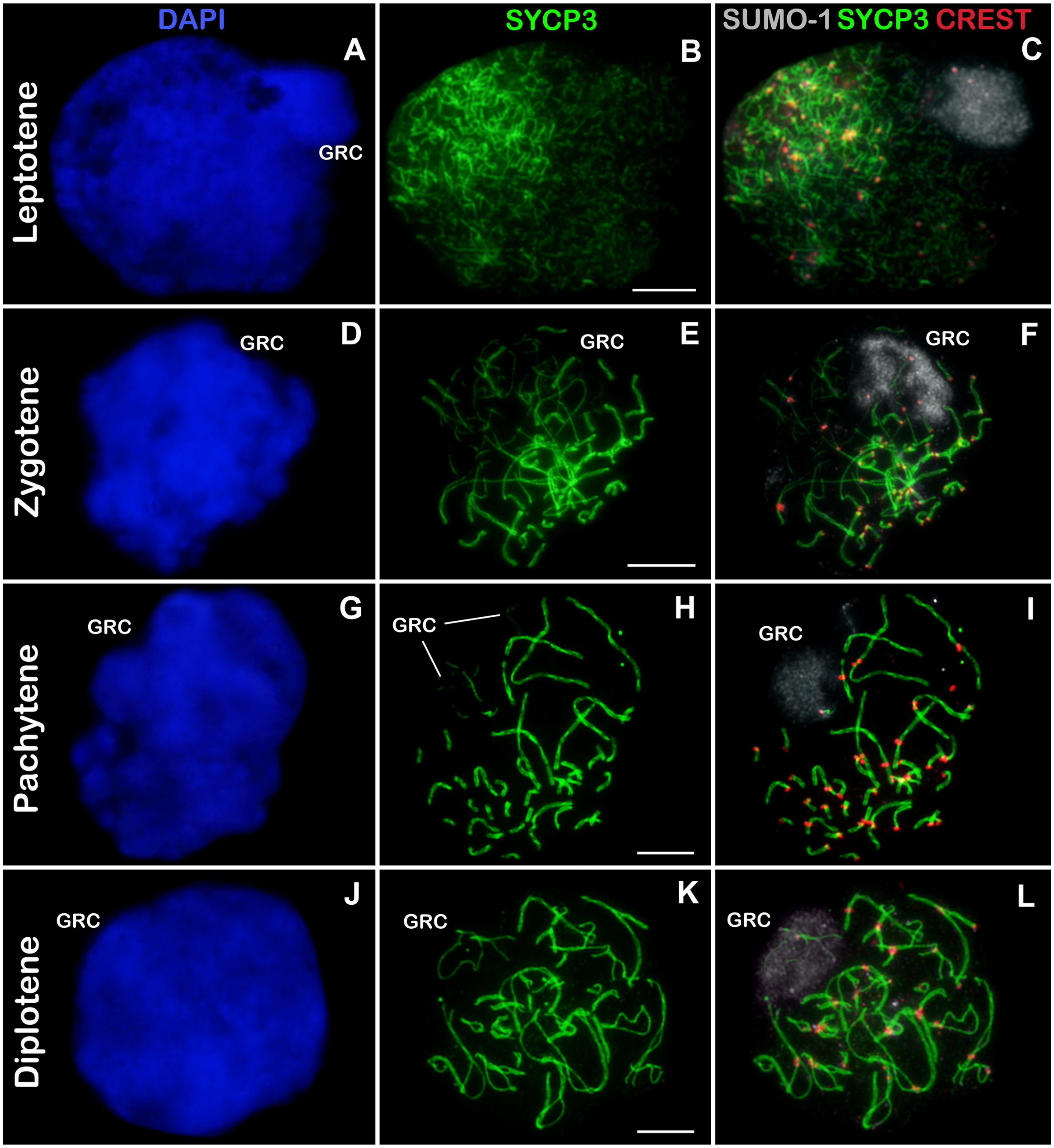

Figure S11. SUMO-1 distribution in zebra finch prophase I spermatocytes. The localizations of SYCP3 (green), CREST (red) and SUMO-1 (grey) are shown. Chromatin is stained by DAPI (blue). GRC – germline-restricted chromosome. **A-C.** Leptotene; **D-F.** Zygotene; **G-I.** Pachytene; **J-L.** Diplotene. Scale bar = 5 μm.

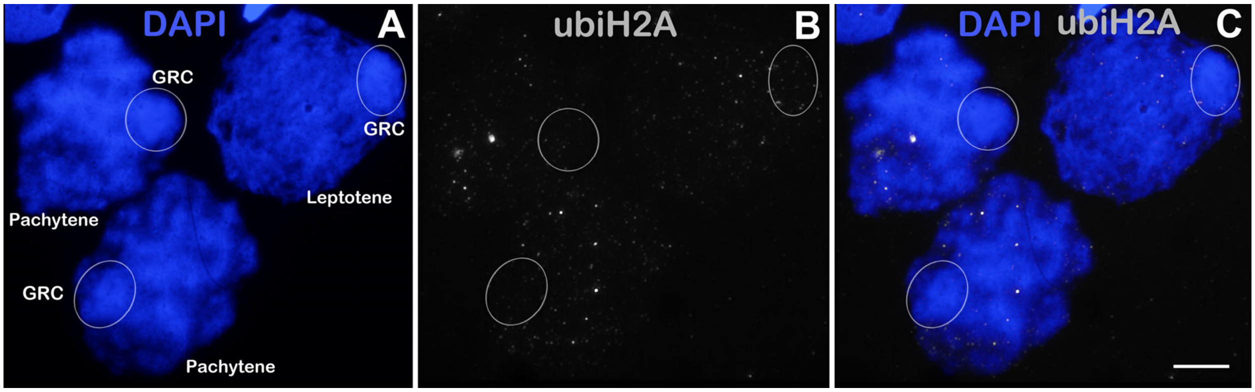

Figure S12. ubiH2A distribution in zebra finch prophase I spermatocytes. The localization of ubiH2A (grey) is shown. Chromatin is stained by DAPI (blue). GRC – germline-restricted chromosome. The white circles point to GRCs. Scale bar = 5 μm.

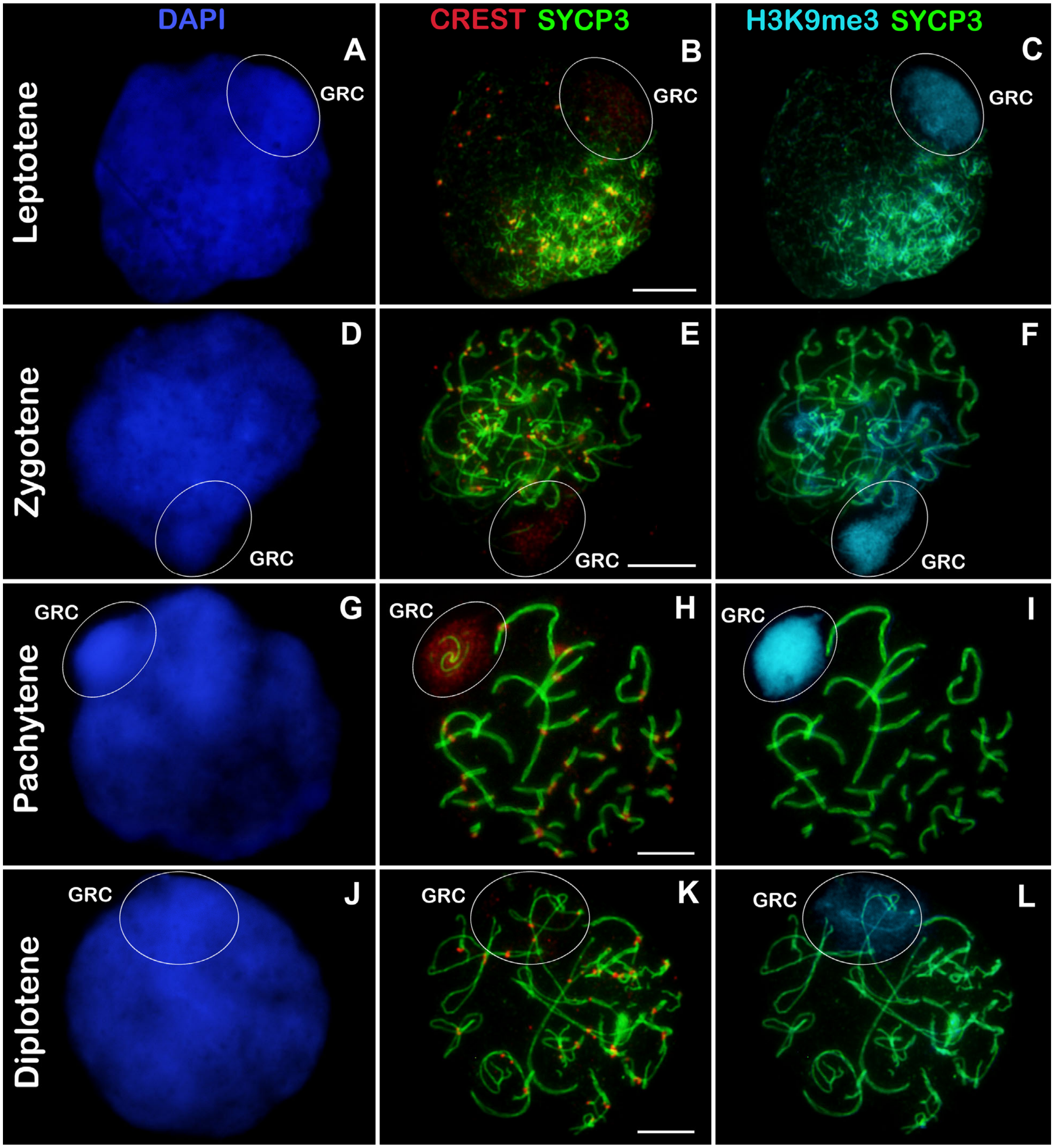

Figure S13. H3K9me3 distribution in zebra finch prophase I spermatocytes. The localizations of SYCP3 (green), CREST (red) and H3K9me3 (cyan) are shown. Chromatin is stained by DAPI (blue). GRC – germline-restricted chromosome. **A-C.** Leptotene; **D-F.** Zygotene; **G-I.** Pachytene; **J-L.** Diplotene. The white circles point to GRCs. Scale bar = 5 µm.

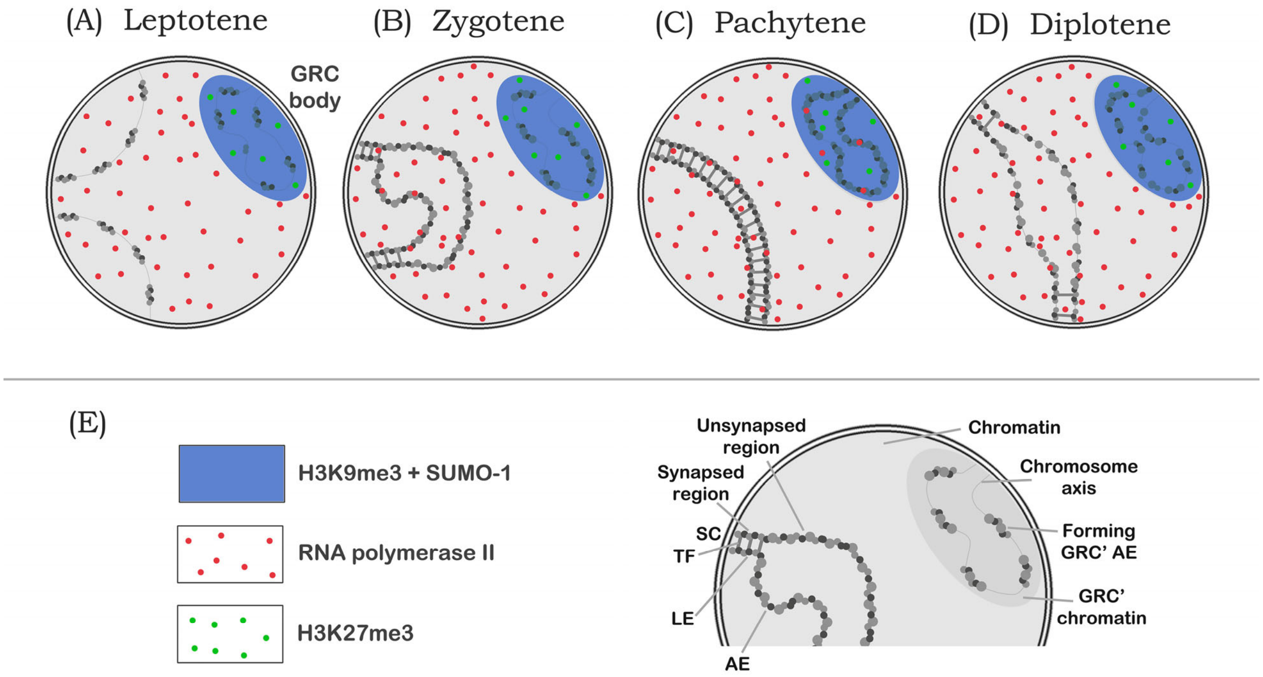

Figure S14. Meiotic silencing of prophase I GRC in zebra finch meiosis. Schematic representations are depicted. Abbreviations: GRC, germline-restricted chromosome; SC, synaptonemal complex; AE, axial element; LE, lateral element of SC; TF, transverse filament of central space of SC. In leptotene (A), zygotene (B), pachytene (C) and diplotene (D), the nuclear chromatin is active (red dots - RNA polymerase II), with the exception of the GRC body. Transcription inactivation in the GRC body was confirmed by the presence of repressive marks (blue color - H3K9me3 and green dots - H3K27me3). In pachytene, the GRC contained several RNAP II dots, implying the presence of transcription sites (C). Explanatory diagrams are presented (E).

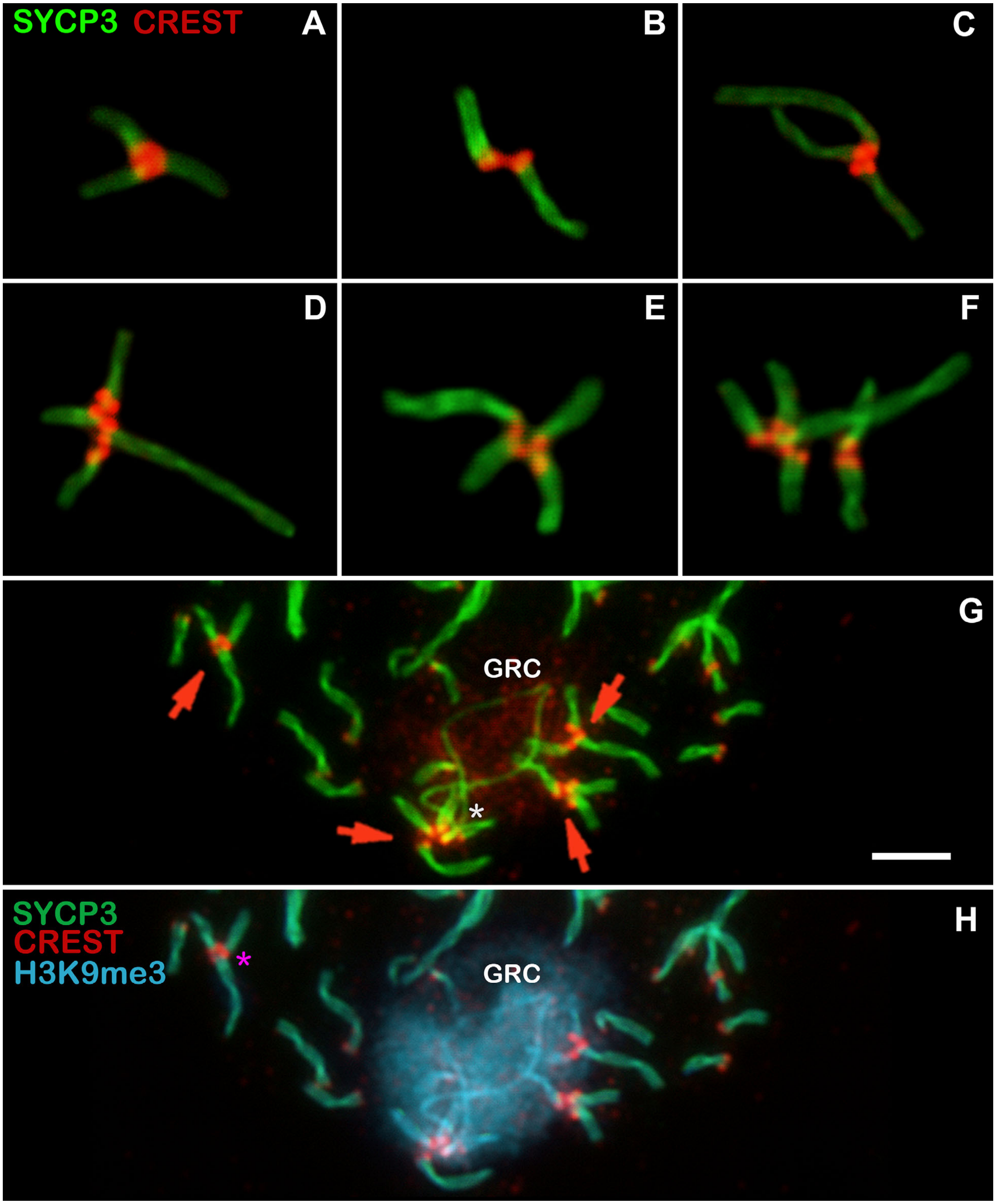

Figure S15. Centromeric association of chromosomes in zebra finch prophase I spermatocytes (A-H). The localizations of SYCP3 (green) and CREST (red) are shown. GRC – germline-restricted chromosome. Red arrows indicate associations of chromosomes via centromeric regions (G). White asterisk indicates possible centromeric association of GRC with microchromosomes (G). Magenta asterisk indicates centromeric association of chromosomes without H3K9me-heterochromatin (H) (similarly for associations on A-F). Scale bar (G) = 5 μm.
